# Role of exponential type random invexities for asymptotically sufficient efficiency conditions in semi-infinite multi-objective fractional programming

**DOI:** 10.1186/s40064-016-3163-8

**Published:** 2016-09-02

**Authors:** Ram U. Verma, Youngsoo Seol

**Affiliations:** 1 Department of Mathematics, University of North Texas, Denton, TX 76201 USA; 2 Department of Mathematics and Statistics, University of South Florida, Tampa, FL 33260 USA

**Keywords:** Semi-infinite programming, Multi-objective fractional programming, Generalized random $(\alpha , \beta , \gamma , \xi , \eta , \rho , h(\cdot , \cdot , \cdot ), \theta )$-invex, Infinitely many equality and inequality constraints, Parametric sufficient efficiency conditions, 90C29, 90C30, 90C32, 90C34, 90C46

## Abstract

First a new notion of the random exponential Hanson–Antczak type $(\alpha ,\beta ,\gamma ,\xi ,\eta ,\rho ,h(\cdot ,\cdot ,\cdot ),\theta )$-V-invexity is introduced, which generalizes most of the existing notions in the literature, second a random function $h(\cdot ,\cdot ,\cdot )$ of the second order is defined, and finally a class of asymptotically sufficient efficiency conditions in semi-infinite multi-objective fractional programming is established. Furthermore, several sets of asymptotic sufficiency results in which various generalized exponential type $HA(\alpha ,\beta ,\gamma ,\xi ,\eta ,\rho ,h(\cdot ,\cdot ,\cdot ),\theta )$-V-invexity assumptions are imposed on certain vector functions whose components are the individual as well as some combinations of the problem functions are examined and proved. To the best of our knowledge, all the established results on the semi-infinite aspects of the multi-objective fractional programming are new, which is a significantly new emerging field of the interdisciplinary research in nature. We also observed that the investigated results can be modified and applied to several special classes of nonlinear programming problems.

## Background

Based on the work of Antczak (2005) on the V-r-invex functions, Zalmai (2013a) generalized and investigated some multi-parameter generalizations of the parametrically sufficient efficiency results under various Hanson–Antczak-type generalized $(\alpha ,\beta ,\gamma ,\xi ,\rho ,\theta )$-V-invexity assumptions for the semi-infinite multi-objective fractional programming problems. Recently, Verma (2013a, 2014) has explored and investigated some results on the multi-objective fractional programming based on new $\epsilon$-optimality conditions, and second-order $(\Phi ,\eta ,\rho ,\theta )$-invexities for parameter-free $\epsilon$-efficiency conditions. Based on the on-going research advances in several areas of multi-objective programming, we observe that the field of the semi-infinite nonlinear multi-objective fractional programming problems seems to be still less explored compared to the general area of mathematical programming. For more details, we refer the readers to Antczak (2005, 2009), Ben-Israel and Mond (1986), Brosowski (1982), Chen and Hu (2009), Craven (1981), Daum and Werner (2011), Ergenç et al. (2004), Fiacco and Kortanek (1983), Giorgi and Guerraggio (1996), Giorgi and Mititelu (1993), Glashoff and Gustafson (1983), Goberna and López (1998, 2001), Gribik (1979), Gustafson and Kortanek (1983), Hanson (1981), Hanson and Mond (1982), Henn and Kischka (1976), Hettich (1976), Hettich and Kortanek (1993), Hettich and Zencke (1982), Jess et al. (2001), Jeyakumar and Mond (1992), Kanniappan and Pandian (1996), López and Still (2007), Martin (1985), Miettinen (1999), Mititelu (2004, 2007), Mititelu and Postolachi (2011), Mititelu and Stancu-Minasian (1993), Mond and Weir (1981), Neralić and Stein (2004), Pini and Singh (1997), Reemtsen and Rückmann (1998), Reiland (1990), Sawaragi et al. (1986), Verma (2013a, b, 2014, 2016), Weber (2002), Weber et al. (2008a, b, 2009), Weber and Tezel (2007), White (1982), Winterfeld (2008), Yu (1985), Zalmai (1998, 2013a, b, c).

In this paper, we plan to introduce the new notion of the random exponential Hanson–Antczak type $(\alpha ,\beta ,\gamma ,\xi ,\eta ,\rho ,h(\cdot ,\cdot ,\cdot ),\theta )$-V-invexity, which generalizes most of the existing notions in the literature, and then establish some results on random function $h(\cdot ,\cdot ,\cdot )$ to the context of a class of asymptotically sufficient efficiency conditions in semi-infinite multi-objective fractional programming.

Now we consider the following semi-infinite multi-objective fractional programming problem based on the random exponential type $HA(\alpha ,\beta ,\gamma ,\xi ,\eta ,\rho ,h(\cdot ,\cdot ,\cdot ),\theta )$-V-invexity:

$$\begin{aligned} (P) \qquad \text{ Minimize } \varphi (x) = \left( \varphi _{1}(x),\ldots ,\varphi _{p}(x)\right) = \left( \dfrac{f_{1}(x)}{g_{1}(x)},\ldots ,\dfrac{f_{p}(x)}{g_{p}(x)}\right) \end{aligned}$$

subject to

1 $$\begin{aligned}&G_{j}(x,t) \leqq 0 \quad \text{for}\;\text{all}\; t \in T_{j},\;\; j \in \underline{q}, \end{aligned}$$

2 $$\begin{aligned}&H_{k}(x,s) = 0\quad \text{for}\;\text{all}\; s \in S_{k},\;\; k \in \underline{r},\nonumber \\&x \in X, \end{aligned}$$

where $p,\; q,$ and *r* are positive integers, *X* is a nonempty open convex subset of $\mathbb {R}^n$ (*n*-dimensional Euclidean space) for each $j \in \underline{q} \equiv \{1, 2, \ldots , q\}$ and $k \in \underline{r},\; T_{j}$ and $S_{k}$ are compact subsets of complete metric spaces for each $i \in \underline{p},\; f_{i}$ and $g_{i}$ are real-valued functions defined on *X*, for each $j \in \underline{q},\; G_{j}(\cdot ,t)$ is a real-valued function defined on *X* for all $t \in T_{j}$, for each $k \in \underline{r},\; H_{k}(\cdot ,s)$ is a real-valued function defined on *X* for all $s \in S_{k}$, for each $j \in \underline{q}$ and $k \in \underline{r},\; G_{j}(x,\cdot )$ and $H_{k}(x,\cdot )$ are continuous real-valued functions defined, respectively, on $T_{j}$ and $S_{k}$ for all $x \in X$, and for each $i \in \underline{p},\; g_{i}(x) > 0$ for all *x* satisfying the constraints of (*P*).

As a matter of fact, all the parametric sufficient efficiency results established in this paper regarding problem (*P*) can easily be modified and restated for each one of the following seven special classes of nonlinear programming problems;

$$\begin{aligned}&(P1)\qquad \underset{x \in \mathbb {F}}{\text{ Minimize}} \left( f_{1}(x) ,\ldots ,f_{p}(x) \right) ;\\&(P2)\qquad \underset{x \in \mathbb {F}}{\text{ Minimize }} \dfrac{f_{1}(x)}{g_{1}(x)};\\&(P3)\qquad \underset{x \in \mathbb {F}}{\text{ Minimize }} f_{1}(x), \end{aligned}$$

where $\mathbb {F}$ (assumed to be nonempty) is the feasible set of (*P*), that is,

3 $$\begin{aligned} \mathbb {F} = \{x \in X : G_{j}(x,t) \leqq 0 \;\; \text{ for}\;\text{all}\;\; t \in T_{j},\; j \in \underline{q},\;\; H_{k}(x,s) = 0\;\; \text{ for}\;\text{all}\;\; s \in S_{k},\;\; k \in \underline{r}\}; \end{aligned}$$

$$\begin{aligned} (P4)\qquad \text{ Minimize} \left( \dfrac{f_{1}(x) }{g_{1}(x) },\ldots ,\dfrac{f_{p}(x) }{g_{p}(x) }\right) \end{aligned}$$

subject to

4 $$\begin{aligned} \tilde{G}_{j}(x) \leqq 0,\;\; j \in \underline{q},\quad \tilde{H}_{k}(x) = 0,\;\; k \in \underline{r},\;\; x \in X, \end{aligned}$$

where $f_{i}$ and $g_{i},\; i \in \underline{p},$ are as defined in the description of $(P),\; \tilde{G_{j}},\; j \in \underline{q}$, and $\tilde{H}_{k},\; k \in \underline{r}$, are real-valued functions defined on *X*;

$$\begin{aligned}&(P5)\qquad \underset{x \in \mathbb {G}}{\text{ Minimize }} \left( f_{1}(x) ,\ldots ,f_{p}(x) \right) ;\\&(P6)\qquad \underset{x \in \mathbb {G}}{\text{ Minimize }} \dfrac{f_{1}(x)}{g_{1}(x)};\\&(P7)\qquad \underset{x \in \mathbb {G}}{\text{ Minimize }} f_{1}(x), \end{aligned}$$

where $\mathbb {G}$ is the feasible set of (*P*4), that is,

5 $$\begin{aligned} \mathbb {G} = \left\{ x \in X : \tilde{G}_{j}(x) \leqq 0, j \in \underline{q},\;\;\tilde{H}_{k}(x) = 0,k \in \underline{r}\right\} . \end{aligned}$$

Furthermore, we introduce the random function $h(\cdot ,\cdot ,\cdot )$ defined on the probability space $(\Omega ,\mathcal {F},P),$ which is the second order and define some new types of invexities regarding randomness and provide some asymptotic sufficient efficiency results for problem (*P*) under various generalized $(\alpha ,\beta ,\gamma ,\xi ,\eta ,\rho ,h(\cdot ,\cdot ,\cdot ),\theta )$-invexity assumptions with the random function $h(\cdot ,\cdot ,\cdot )$.

The rest of the paper is organized as follows. Some introductory and basic concepts are introduced and studied in “Preliminaries” section along with introduction of the exponential type $HA(\alpha ,\beta ,\gamma ,\xi ,\eta ,\rho , h(\cdot ,\cdot ,\cdot ),\theta )$-V-invexities under the random function $h(\cdot ,\cdot ,\cdot ),$ which generalizes $HA(\alpha ,\beta ,\gamma ,\xi ,\eta ,\rho ,h(\cdot ,\cdot ), \theta )$-V-invexities. In “Asymptotic sufficiency conditions” section, we discuss some sufficient efficiency conditions where we formulate and prove several sets of sufficiency criteria under a variety of the exponential type $HA(\alpha ,\beta ,\gamma ,\xi ,\eta ,\rho , h(\cdot ,\cdot ,\cdot ),\theta )$-V-invexities with the random function $h(\cdot ,\cdot ,\cdot )$ that are placed on certain vector-valued functions whose entries consist of the individual as well as some combinations of the problem functions. “Concluding remarks” section concludes the paper with final remarks on the obtained results and their future applications to other fields of research.

## Preliminaries

In this section, we first introduce the concepts of the general probability theory and the exponential type $HA(\alpha ,\beta ,\gamma ,\xi ,\eta ,\rho ,h(\cdot ,\cdot ,\cdot ),\theta )$-V-invexities under the random function $h(\cdot ,\cdot ,\cdot )$, and then recall some other related auxiliary results instrumental to the problem on hand.

### Random variables and probability theory

In this subsection, we review the fundamental concepts of the probability theory on which the function $h(\cdot ,\cdot ,\cdot )$ can be defined. We first define here the probability space and filtered probability space as the followings;

#### **Definition 1**

A probability space is a triple $(\Omega ,\mathcal {F},P),$ where

- $\Omega$ is a set of all events which is called sample space. Elements of $\Omega$ are denoted by $\omega$ and are sometimes called outcomes.
- $\mathcal {F}$ is a $\sigma$-algebra (or $\sigma$-field), i.e., a nonempty collection of subsets of $\Omega$ that satisfy
  (i) if $A\in \mathcal {F}$ then $A^{c}\in \mathcal {F},$ and
  (ii) if $A_{i}\in \mathcal {F}$ is a countable sequence of sets then $\cap _{i}A_{i}\in \mathcal {F}.$
- P: $\mathcal {F}\rightarrow [0,1]$ is a function with $P(\Omega )=1$ and such that if $E_{1},E_{2},\ldots \in \mathcal {F}$ are disjoint,
  6 $$\begin{aligned} P\left( \bigcap \limits _{j=1}^{\infty }E_{j}\right) =\sum _{j=1}^{\infty }P(E_{j}). \end{aligned}$$

#### **Definition 2**

Let $$(\Omega ,\mathcal {F},P)$$ be a probability space. A filtration on $$(\Omega ,\mathcal {F},P)$$ is an increasing family $$(\mathcal {F}_{t})_{t\ge 0}$$ of $$\sigma$$-algebra of $$\mathcal {F}.$$ In other words, for each t, $$\mathcal {F}_{t}$$ is a $$\sigma$$-algebra included in $$\mathcal {F}$$ and if $$s\le t,$$$$\mathcal {F}_{s}\le \mathcal {F}_{t}.$$ A probability space $$(\Omega ,\mathcal {F},P)$$ endowed with a filtration $$(\mathcal {F}_{t})_{t\ge 0}$$ is called a filtrated probability space.

Filtration have been a feature of the theory in the literature of the stochastic processes and mathematical fiance and advanced probability field such as stochastic control theory, martingales, semi-martingales, stopping times or Markov processes. In this paper, we restrict the concept filtrated probability space to investigate some results regarding the function h as defined by the martingale processes or Markov chain in the future work. In the followings, we define the random variables and study the concepts of the first and second moments of random variables.

#### **Definition 3**

A random variable X is a measurable function from a probability space $(\Omega ,\mathcal {F},P)$ to the reals, i.e., it is a function

7 $$\begin{aligned} X:\Omega \rightarrow (-\infty ,\infty ) \end{aligned}$$

such that for every Borel set B,

8 $$\begin{aligned} X^{-1}(B)=\{\omega \in \Omega :X(\omega )\in B\}\in \mathcal {F}. \end{aligned}$$

Furthermore, we define a function on Borel sets by

9 $$\begin{aligned} \mu _{X}(B)=P\{X\in B\}=P[X^{-1}(B)]. \end{aligned}$$

Let X be a random variable. We define the expectation of X, denoted by $\mathbb {E}(X),$ by

10 $$\begin{aligned} \mathbb {E}(X)=\int X dP, \end{aligned}$$

where the integral is the Lebesgue integral. In particular, the expectation of a random variable depends only on its distribution and not on the probability space on which it is defined. If X has a density F, then the measure $\mu _{X}$ is the same as *f*(*x*)*dx*, so we can write

11 $$\begin{aligned} \mathbb {E}[X]=\int _{-\infty }^{\infty }xf(x) dx, \end{aligned}$$

where again the expectation exists if and only if

12 $$\begin{aligned} \int _{-\infty }^{\infty }|x|f(x) dx<\infty . \end{aligned}$$

Furthermore, the second moment is defined by

13 $$\begin{aligned} \mathbb {E}[X^{2}]=\int _{-\infty }^{\infty }x^{2}f(x) dx,~\text{ and }~Var[X]=\mathbb {E}[X-\mathbb {E}(X)]=\mathbb {E}[X^{2}]-(\mathbb {E}[X])^{2}. \end{aligned}$$

The following are the concepts of independence.

#### **Definition 4**

- $\sigma$-algebra $\mathcal {F}_{1},\mathcal {F}_{2},\ldots ,\mathcal {F}_{n}$ are independent if whenever $A_{i}\in \mathcal {F}_{i}$ for $i=1,\ldots ,n$ we have
  14 $$\begin{aligned} P\left( \bigcap \limits _{i=1}^{n}A_{i}\right) =\prod _{i=1}^{n}P(A_{i}). \end{aligned}$$
- Random variables $X_{1},X_{2},\ldots ,X_{n}$ are independent if for every Borel sets $B_{i}$ for $i=1,\ldots ,n$ we have
  15 $$\begin{aligned} P\left( \bigcap \limits _{i=1}^{n}\{X_{i}\in B_{i}\}\right) =\prod _{i=1}^{n}P(X_{i}\in B_{i}). \end{aligned}$$

The law of large numbers, which is a theorem proved about the mathematical model of probability, shows that this model is consistent with the frequency interpretation of probability and this theorem is the main idea to prove the main theorem in this paper.

#### **Theorem 5**

(Law of Large Numbers) *Let*$X_{1},X_{2},\ldots ,X_{n}$*be a sequence of independent random variables with common distribution function.Set*$\mu =\mathbb {E}[X_{i}]<\infty$*and*$\sigma ^{2}=Var[X_{i}],$*and for*$S_{n}=\sum \nolimits _{i=1}^{n}X_{i},$*we have *

(a): *Weak law of large numbers*
  16 $$\begin{aligned} \lim _{n\rightarrow \infty }P\left( \omega :\left| \frac{S_{n}(\omega )}{n}-\mu \right| \ge \epsilon \right) =0,\quad \forall \epsilon >0. \end{aligned}$$
(b): *Strong law of large numbers*
  17 $$\begin{aligned} P\left( \omega :\lim _{n\rightarrow \infty }\frac{S_{n}(\omega )}{n}=\mu \right) =1. \end{aligned}$$

### Deterministic cases

#### **Definition 6**

Let *f* be a differentiable real-valued function defined on $\mathbb {R}^n$. Then *f* is said to be $\eta$-invex (invex with respect to $\eta$) at $y\in \mathbb {R}^n$ if there exists a function $\eta : \mathbb {R}^n \times \mathbb {R}^n \rightarrow \mathbb {R}^{n}$ such that for each $x \in \mathbb {R}^n$,

18 $$\begin{aligned} f(x) - f(y) \geqq \langle \nabla f(y),\eta (x,y) \rangle , \end{aligned}$$

where $\nabla f(y) = (\partial f(y)/\partial y_{1}, \partial f(y)/\partial y_{2}, \ldots , \partial f(y)/\partial y_{n})$ is the gradient of *f* at *y*, and $\langle a,b \rangle$ denotes the inner product of the vectors *a* and $b; \; f$ is said to be $\eta$-invex on $\mathbb {R}^n$ if the above inequality holds for all $x, y \in \mathbb {R}^n$.

Hanson (1981) showed (based on the role of the function $\eta$) that for a nonlinear programming problem of the form

$$\begin{aligned} \text{ Minimize }\;f(x) \quad \text{ subject } \text{ to }\; g_{i}(x) \leqq 0,\quad i \in \underline{m},\;\; x \in \mathbb {R}^{n}, \end{aligned}$$

where the differentiable functions $f, g_{i} : \mathbb {R}^{n} \rightarrow \mathbb {R},\,i \in \underline{m}$, are invex with respect to the function $\eta : \mathbb {R}^{n}\times \mathbb {R}^{n}\rightarrow \mathbb {R}^{n}$,  the Karush–Kuhn–Tucker necessary optimality conditions are also sufficient.

Let the function $F=(F_1,F_2,\ldots ,F_N):\mathbb {R}^n \rightarrow \mathbb {R}^{N}$ be differentiable at $x^{*}$. The following generalizations of the notions of invexity, pseudoinvexity, and quasiinvexity for vector-valued functions were originally proposed in Jeyakumar and Mond (1992).

#### **Definition 7**

The function *F* is said to be $(\alpha ,\eta )$*-V-invex at*$x^{*}$ if there exist functions $\alpha _{i} : \mathbb {R}^n \times \mathbb {R}^n \rightarrow \mathbb {R}_{+}\backslash \{0\}\equiv (0,\infty ),\; i \in \underline{N}$, and $\eta : \mathbb {R}^n \times \mathbb {R}^n \rightarrow \mathbb {R}^{n}$ such that for each $x \in \mathbb {R}^n$ and $i \in \underline{N}$,

19 $$\begin{aligned} F_{i}(x) - F_{i}(x^{*}) \geqq \left\langle \alpha _{i}(x,x^{*})\nabla F_{i}(x^{*}),\eta (x,x^{*})\right\rangle . \end{aligned}$$

#### **Definition 8**

The function *F* is said to be $(\beta ,\eta )$*-V-pseudoinvex at*$x^{*}$ if there exist functions $\beta _{i} : \mathbb {R}^n \times \mathbb {R}^n \rightarrow \mathbb {R}_{+}\backslash \{0\}, \; i \in \underline{N}$, and $\eta : \mathbb {R}^n \times \mathbb {R}^n \rightarrow \mathbb {R}^{n}$ such that for each $x \in \mathbb {R}^n$,

20 $$\begin{aligned} \left\langle \sum _{i=1}^{N}\nabla F_{i}(x^{*}), \eta (x,x^{*})\right\rangle \geqq 0 \Rightarrow \sum _{i=1}^{N}\beta _{i}(x,x^{*})F_{i}(x) \geqq \sum _{i=1}^{N}\beta _{i}(x,x^{*})F_{i}(x^{*}). \end{aligned}$$

#### **Definition 9**

The function *F* is said to be $(\gamma ,\eta )$*-V-quasiinvex at*$x^{*}$ if there exist functions $\gamma _{i} : \mathbb {R}^n \times \mathbb {R}^n \rightarrow \mathbb {R}_{+}\backslash \{0\}, \; i \in \underline{N}$, and $\eta : \mathbb {R}^n \times \mathbb {R}^n \rightarrow \mathbb {R}^{n}$ such that for each $x \in \mathbb {R}^n$,

21 $$\begin{aligned} \sum _{i=1}^{N}\gamma _{i}(x,x^{*})F_{i}(x) \leqq \sum _{i=1}^{N}\gamma _{i}(x,x^{*})F_{i}(x^{*}) \Rightarrow \left\langle \sum _{i=1}^{N}\nabla F_{i}(x^{*}), \eta (x,x^{*})\right\rangle \leqq 0. \end{aligned}$$

Recently, Antczak (2005) introduced the following exponential type of the class of V-invex functions.

#### **Definition 10**

A differentiable function $f : X \rightarrow \mathbb {R}^{k}$ is called (strictly) $\zeta _{i}$-$\tilde{r}$-invex with respect to $\eta$ at $u \in X$ if there exist functions $\eta : X\times X \rightarrow \mathbb {R}^{n}$ and $\zeta _{i} : X\times X \rightarrow \mathbb {R}_{+}\backslash \{0\},\; i \in \underline{k}$, such for each $x \in X$,

22 $$\begin{aligned}&\frac{1}{\tilde{r}}e^{\tilde{r}f_{i}(x)} ({>}) \geqq \frac{1}{\tilde{r}}e^{\tilde{r}f_{i}(u)}[1 + \tilde{r}\zeta _{i}(x,u)\langle \nabla f_{i}(u),\eta (x,u)\rangle ] \;\; \text{ for } \tilde{r} \ne 0, \end{aligned}$$

23 $$\begin{aligned}&f_{i}(x) - f_{i}(u) \geqq \zeta _{i}(x,u)\langle \nabla f_{i}(u),\eta (x,u)\rangle \quad \text{ for } \tilde{r} = 0. \end{aligned}$$

As the exponential type of the class of functions was considered in Antczak (2005) for establishing some sufficiency and duality results for a nonlinear programming problem with differentiable functions, and their nonsmooth analogues were discussed in Antczak (2009), recently, Zalmai (2013a) introduced the Hanson–Antczak type generalized $HA(\alpha ,\beta ,\gamma ,\xi ,\eta ,\rho ,\theta )$-V-invexity, an exponential type framework, and then applied to a set of problems on fractional programming. As a result, Zalmai further envisioned a vast array of interesting and significant classes of generalized convex functions. Now we are ready to present the exponential type $HA(\alpha ,\beta ,\gamma ,\xi ,\eta ,\rho ,h(\cdot ,\cdot ),\theta )$-V-invexities that generalize and encompass most of the existing notions available in the current literature. Let the function $F = (F_{1},F_{2},\ldots ,F_{p}):X \rightarrow \mathbb {R}^{p}$ be differentiable at $x^{*}$.

#### **Definition 11**

The function *F* is said to be (strictly) $HA(\alpha ,\beta ,\gamma ,\xi ,\eta ,\rho ,h(\cdot ,\cdot ),\theta )$-V-invex at $x^{*}\in X$ if there exist functions $\alpha : X\times X \rightarrow \mathbb {R},\;\beta : X\times X \rightarrow \mathbb {R},\; \gamma _{i} : X\times X \rightarrow \mathbb {R}_{+}, \; \xi _{i} : X\times X \rightarrow \mathbb {R}_{+}\backslash \{0\},\; i \in \underline{p},\; z\in \mathbb {R}^{n}, \eta :X\times X\rightarrow \mathbb {R}^n, \rho _{i} : X\times X \rightarrow \mathbb {R},\; i \in \underline{p}$, and $\theta : X\times X \rightarrow \mathbb {R}^{n}$ such that for all $x \in X \;(x \ne x^{*})$ and $i \in \underline{p}$,

24 $$\begin{aligned}&\frac{1}{\alpha (x,x^{*})}\gamma _{i}(x,x^{*})\left( e^{\alpha (x,x^{*})\left[ F_{i}(x) - F_{i}(x^{*})\right] } - 1\right) \nonumber \\&\quad ({>}) \geqq \frac{1}{\beta (x,x^{*})}\left\langle \xi _{i}(x,x^{*})\nabla _{z}h_i(x^{*},z),e^{\beta (x,x^{*})\eta (x,x^*)} - \mathbf {1}\right\rangle \nonumber \\&\quad\quad\qquad +\rho _{i}(x,x^{*})\Vert \theta (x,x^{*})\Vert ^{2}\quad \text{ if } \alpha (x,x^{*})\ne 0 \text{ and } \beta (x,x^{*})\ne 0 \;\;\text{ for}\;\text{all}\; x \in X, \end{aligned}$$

25 $$\begin{aligned}&\frac{1}{\alpha (x,x^{*})}\gamma _{i}(x,x^{*})\left( e^{\alpha (x,x^{*})\left[ F_{i}(x) - F_{i}(x^{*})\right] } - 1\right) ({>}) \geqq \left\langle \xi _{i}(x,x^{*})\nabla _{z}h_i(x^{*},z),\eta (x,x^*)\right\rangle \nonumber \\&\quad+ \rho _{i}(x,x^{*})\Vert \theta (x,x^{*})\Vert ^{2} \quad \text{ if } \alpha (x,x^{*}) \ne 0 \text{ and } \beta (x,x^{*})\rightarrow 0 \;\;\text{ for}\;\text{all}\; x \in X, \end{aligned}$$

26 $$\begin{aligned}&\gamma _{i}(x,x^{*})\left[ F_{i}(x) - F_{i}(x^{*})\right] ({>}) \geqq \frac{1}{\beta (x,x^{*})}\left\langle \xi _{i}(x,x^{*})\nabla _{z}h_i(x^{*},z),e^{\beta (x,x^{*})\eta (x,x^*)} - \mathbf {1}\right\rangle \nonumber \\&\quad +\rho _{i}(x,x^{*})\Vert \theta (x,x^{*})\Vert ^{2} \quad \text{ if } \alpha (x,x^{*})\rightarrow 0 \text{ and } \beta (x,x^{*}) \ne 0 \;\;\text{ for}\;\text{all}\; x \in X, \end{aligned}$$

27 $$\begin{aligned}\gamma _{i}(x,x^{*})\left[ F_{i}(x) - F_{i}(x^{*})\right] ({>}) &\geqq \left\langle \xi _{i}(x,x^{*})\nabla _{z}h_i(x^{*},z),\eta (x,x^*) \right\rangle + \rho _{i}(x,x^{*})\Vert \theta (x,x^{*})\Vert ^{2} \nonumber \\&\quad \text{ if } \alpha (x,x^{*})\rightarrow 0 \text{ and } \beta (x,x^{*}) \rightarrow 0 \;\;\text{ for}\;\text{all}\; x \in X, \end{aligned}$$

where $\Vert \cdot \Vert$ is a norm on $\mathbb {R}^{n}$ and

28 $$\begin{aligned} \left( e^{\beta (x,x^{*})\eta _(x,x^*)} - \mathbf {1}\right) \equiv \left( e^{\beta (x,x^{*})\eta _{1}(x,x^*)} - 1,\ldots ,e^{\beta (x,x^{*})\eta _{n}(x,x^*)} - 1\right) , \end{aligned}$$

with $$h:\mathbb {R}^n\times \mathbb {R}^n \rightarrow \mathbb {R}^n$$ differentiable.

#### **Definition 12**

The function *F* is said to be (strictly) $HA(\alpha ,\beta ,\gamma ,\xi ,\eta ,\rho ,h(\cdot ,\cdot ),\theta )$-V-pseudoinvex at $x^{*}\in X$ if there exist functions $\alpha : X\times X \rightarrow \mathbb {R}, \; \beta : X\times X \rightarrow \mathbb {R}, \; \gamma : X\times X \rightarrow \mathbb {R}_{+}, \; \xi _{i} : X\times X \rightarrow \mathbb {R}_{+}\backslash \{0\}, \; i \in \underline{p}, \; z \in \mathbb {R}^{n}, \; \eta :X\times X\rightarrow \mathbb {R}^n$, $\rho : X\times X \rightarrow \mathbb {R}$, and $\theta : X\times X \rightarrow \mathbb {R}^{n}$ such that for all $x \in X\; (x \ne x^{*})$,

29 $$\begin{aligned}&\frac{1}{\beta (x,x^{*})}\left\langle \sum _{i=1}^{p}\nabla _z h_i(x^*,z),e^{\beta (x,x^{*})\eta (x,x^*))} - \mathbf {1}\right\rangle \geqq - \rho (x,x^{*})\Vert \theta (x,x^{*})\Vert ^{2} \nonumber \\&\quad \Rightarrow \frac{1}{\alpha (x,x^{*})}\gamma (x,x^{*})\left( e^{\alpha (x,x^{*})\sum _{i=1}^{p}\xi _{i}(x,x^{*})\left[ F_{i}(x) - F_{i}(x^{*})\right] } - 1\right) ({>}) \geqq 0 \nonumber \\&\qquad \text{ if } \alpha (x,x^{*})\ne 0 \text{ and } \beta (x,x^{*})\ne 0 \;\;\text{ for}\;\text{all}\; x \in X, \end{aligned}$$

30 $$\begin{aligned}&\left\langle \sum _{i=1}^{p}\nabla _z h_{i}(x^{*},z),\eta (x,x^{*})\right\rangle \geqq - \rho (x,x^{*})\Vert \theta (x,x^{*})\Vert ^{2} \nonumber \\&\quad \Rightarrow \frac{1}{\alpha (x,x^{*})}\gamma (x,x^{*})\left( e^{\alpha (x,x^{*})\sum _{i=1}^{p}\xi _{i}(x,x^{*})\left[ F_{i}(x) - F_{i}(x^{*})\right] } - 1\right) ({>}) \geqq 0 \nonumber \\&\qquad \text{ if } \alpha (x,x^{*}) \ne 0 \text{ and } \beta (x,x^{*}) \rightarrow 0 \;\;\text{ for}\;\text{all}\; x \in X, \end{aligned}$$

31 $$\begin{aligned}&\frac{1}{\beta (x,x^{*})}\left\langle \sum _{i=1}^{p}\nabla _z h_{i}(x^{*},z),e^{\beta (x,x^{*})\eta (x,x^{*})} - \mathbf {1}\right\rangle \geqq - \rho (x,x^{*})\Vert \theta (x,x^{*})\Vert ^{2} \nonumber \\&\quad \Rightarrow \gamma (x,x^{*})\sum _{i=1}^{p}\xi _{i}(x,x^{*})\left[ F_{i}(x) - F_{i}(x^{*})\right] ({>}) \geqq 0 \nonumber \\&\qquad \text{ if } \alpha (x,x^{*}) \rightarrow 0 \text{ and } \beta (x,x^{*}) \ne 0\; \text{ for}\;\text{all}\; x \in X, \end{aligned}$$

32 $$\begin{aligned}&\left\langle \sum _{i=1}^{p}\nabla _z h_{i}(x^{*},z),\eta (x,x^{*})\right\rangle \geqq - \rho (x,x^{*})\Vert \theta (x,x^{*})\Vert ^{2} \nonumber \\&\quad \Rightarrow \gamma (x,x^{*})\sum _{i=1}^{p}\xi _{i}(x,x^{*})\left[ F_{i}(x) - F_{i}(x^{*})\right] ({>}) \geqq 0 \nonumber \\&\qquad \text{ if } \alpha (x,x^{*}) \rightarrow 0 \text{ and } \beta (x,x^{*}) \rightarrow 0 \;\;\text{ for}\;\text{all}\; x \in X. \end{aligned}$$

with $h:\mathbb {R}^n\times \mathbb {R}^n \rightarrow \mathbb {R}^n$ differentiable. The function *F* is said to be (strictly) $HA(\alpha ,\beta ,\gamma ,\xi ,\eta ,\rho ,h(\cdot ,\cdot ),\theta )$-V-pseudoinvex on *X* if it is (strictly) $HA(\alpha ,\beta ,\gamma ,\xi ,\eta ,\rho ,h(\cdot ,\cdot ),\theta )$-V-pseudoinvex at each point $x^{*} \in X$.

#### **Definition 13**

The function *F* is said to be (prestrictly) $HA(\alpha ,\beta ,\gamma ,\xi ,\eta ,\rho ,h(\cdot ,\cdot ),\theta )$-V-quasiinvex at $x^{*}\in X$ if there exist functions $\alpha : X\times X \rightarrow \mathbb {R}, \; \beta : X\times X \rightarrow \mathbb {R},\; \gamma : X\times X \rightarrow \mathbb {R}_{+},\; \xi _{i} : X\times X \rightarrow \mathbb {R}_{+}\backslash \{0\}, \; i \in \underline{p},\; \eta : X\times X \rightarrow \mathbb {R}^{n},\; \rho : X\times X \rightarrow \mathbb {R}$, and $\theta : X\times X \rightarrow \mathbb {R}^{n}$ such that for all $x \in X$,

33 $$\begin{aligned}&\frac{1}{\alpha (x,x^{*})}\gamma (x,x^{*})\left( e^{\alpha (x,x^{*})\sum _{i=1}^{p}\xi _{i}(x,x^{*})\left[ F_{i}(x) - F_{i}(x^{*})\right] } - 1\right) ({<}) \leqq 0 \nonumber \\&\quad \Rightarrow \frac{1}{\beta (x,x^{*})}\left\langle \sum _{i=1}^{p}\nabla _z h_{i}(x^{*},z),e^{\beta (x,x^{*})\eta (x,x^{*})} - \mathbf {1}\right\rangle \leqq - \rho (x,x^{*})\Vert \theta (x,x^{*})\Vert ^{2} \nonumber \\&\qquad \text{ if } \alpha (x,x^{*})\ne 0 \text{ and } \beta (x,x^{*})\ne 0 \;\;\text{ for}\;\text{all}\; x \in X, \end{aligned}$$

34 $$\begin{aligned}&\frac{1}{\alpha (x,x^{*})}\gamma (x,x^{*})\left( e^{\alpha (x,x^{*})\sum _{i=1}^{p}\xi _{i}(x,x^{*})\left[ F_{i}(x) - F_{i}(x^{*})\right] } - 1\right) ({<}) \leqq 0 \nonumber \\&\quad \Rightarrow \left\langle \sum _{i=1}^{p}\nabla _z h_{i}(x^{*},z),\eta (x,x^{*})\right\rangle \leqq - \rho (x,x^{*})\Vert \theta (x,x^{*})\Vert ^{2} \nonumber \\&\qquad \text{ if } \alpha (x,x^{*}) \ne 0 \text{ and } \beta (x,x^{*}) \rightarrow 0 \;\;\text{ for}\;\text{all}\; x \in X, \end{aligned}$$

35 $$\begin{aligned}&\gamma (x,x^{*})\sum _{i=1}^{p}\xi _{i}(x,x^{*})\left[ F_{i}(x) - F_{i}(x^{*})\right] ({<}) \leqq 0 \nonumber \\&\quad \Rightarrow \frac{1}{\beta (x,x^{*})}\left\langle \sum _{i=1}^{p}\nabla _z h_{i}(x^{*},z),e^{\beta (x,x^{*})\eta (x,x^{*})} - \mathbf {1}\right\rangle \leqq - \rho (x,x^{*})\Vert \theta (x,x^{*})\Vert ^{2} \nonumber \\&\qquad \text{ if } \alpha (x,x^{*}) \rightarrow 0 \text{ and } \beta (x,x^{*}) \ne 0 \;\;\text{ for}\;\text{all}\; x \in X, \end{aligned}$$

36 $$\begin{aligned}&\gamma (x,x^{*})\sum _{i=1}^{p}\xi _{i}(x,x^{*})\left[ F_{i}(x) - F_{i}(x^{*})\right] ({<}) \leqq 0 \nonumber \\&\quad \Rightarrow \left\langle \sum _{i=1}^{p}\nabla _z h_{i}(x^{*},z),\eta (x,x^{*}) \right\rangle \leqq - \rho (x,x^{*})\Vert \theta (x,x^{*})\Vert ^{2} \nonumber \\&\qquad \text{ if } \alpha (x,x^{*}) \rightarrow 0 \text{ and } \beta (x,x^{*}) \rightarrow 0 \;\;\text{ for}\;\text{all}\; x \in X. \end{aligned}$$

with $$h:\mathbb {R}^n\times \mathbb {R}^n \rightarrow \mathbb {R}^n$$ differentiable.

#### *Example 14*

The function *F* is said to be (strictly) $HA(\alpha ,\beta ,\gamma ,\xi ,\eta ,\rho ,\theta )$-V-invex at $x^{*}\in X$ if there exist functions $\alpha : X\times X \rightarrow \mathbb {R},\;\beta : X\times X \rightarrow \mathbb {R},\; \gamma _{i} : X\times X \rightarrow \mathbb {R}_{+}, \; \xi _{i} : X\times X \rightarrow \mathbb {R}_{+}\backslash \{0\},\; i \in \underline{p},\; z\in \mathbb {R}^{n}, \eta :X\times X\rightarrow \mathbb {R}^n,\; \rho _{i} : X\times X \rightarrow \mathbb {R},\; i \in \underline{p}$, and $\theta : X\times X \rightarrow \mathbb {R}^{n}$ such that for all $x \in X \;(x \ne x^{*})$ and $i \in \underline{p}$,

37 $$\begin{aligned}&\frac{1}{\alpha (x,x^{*})}\gamma _{i}(x,x^{*})\left( e^{\alpha (x,x^{*})\left[ F_{i}(x) - F_{i}(x^{*})\right] } - 1\right) \nonumber \\&\quad ({>}) \geqq \frac{1}{\beta (x,x^{*})}\left\langle \xi _{i}(x,x^{*})\nabla f(x^{*}), e^{\beta (x,x^{*})\eta (x,x^*)} - \mathbf {1}\right\rangle +\rho _{i}(x,x^{*})\Vert \theta (x,x^{*})\Vert ^{2}\;\;\;\nonumber \\&\qquad \text{ if } \alpha (x,x^{*})\ne 0 \text{ and } \beta (x,x^{*})\ne 0 \;\;\text{ for}\;\text{all}\; x \in X. \end{aligned}$$

We also noticed that for the proofs of the sufficient efficiency theorems, sometimes it may be more appropriate to apply certain alternative but equivalent forms of the above definitions based on considering the contrapositive statements. For example, the exponential type $HA(\alpha ,\beta ,\gamma ,\xi ,\eta ,\rho ,h(\cdot ,\cdot ),\theta )$-V-quasiinvexity (when $\alpha (x,x^{*})\ne 0$ and $\beta (x,x^{*})\ne 0$ for all $x \in X$) can be defined in the following equivalent way:

The function *F* is an exponential type $HA(\alpha ,\beta ,\gamma ,\xi ,\eta ,\rho ,h(\cdot ,\cdot ),\theta )$-V-quasiinvex at $x^{*}\in X$ if there exist functions $\alpha : X\times X \rightarrow \mathbb {R}, \;\beta : X\times X \rightarrow \mathbb {R}, \; \gamma : X\times X \rightarrow \mathbb {R}_{+},\; \xi _{i} : X\times X \rightarrow \mathbb {R}_{+}\backslash \{0\}, \; i \in \underline{p},\; \eta : X\times X \rightarrow \mathbb {R}^{n}, \; \rho : X\times X \rightarrow \mathbb {R}$, and $\theta : X\times X \rightarrow \mathbb {R}^{n}$ such that for all $x \in X$,

38 $$\begin{aligned}&\frac{1}{\beta (x,x^{*})}\left\langle \sum _{i=1}^{p}\nabla _z h_{i}(x^{*},z),e^{\beta (x,x^{*})\eta (x,x^{*})} - \mathbf {1}\right\rangle> - \rho (x,x^{*})\Vert \theta (x,x^{*})\Vert ^{2}\nonumber \\&\quad \Rightarrow \frac{1}{\alpha (x,x^{*})}\gamma (x,x^{*})\left( e^{\alpha (x,x^{*})\sum _{i=1}^{p}\xi _{i}(x,x^{*})\left[ F_{i}(x) - F_{i}(x^{*})\right] } - 1\right) > 0, \end{aligned}$$

where $h:\mathbb {R}^n\times \mathbb {R}^n \rightarrow \mathbb {R}^n$ is differentiable.

In the sequel, we shall also need a consistent notation for vector inequalities. For $a, b \in \mathbb {R}^{m}$, the following order notation will be used: $a \geqq b$ if and only if $a_{i} \geqq b_{i}$ for all $i \in \underline{m}$; $a \geqslant b$ if and only if $a_{i} \geqq b_{i}$ for all $i \in \underline{m}$, but $a \ne b$; $a > b$ if and only if $a_{i} > b_{i}$ for all $i \in \underline{m}$; and $a \ngeqslant b$ is the negation of $a \geqslant b$.

Consider the multi-objective problem

$$\begin{aligned} (P^{*}) \qquad \underset{x \in \mathbb {F}}{\text{ Minimize }} \; F(x) = (F_{1}(x),\ldots ,F_{p}(x)), \end{aligned}$$

where $F_{i},\; i \in \underline{p}$, are real-valued functions defined on $\mathbb {R}^n$.

An element $x^{\circ } \in \mathbb {F}$ is said to be an *efficient* (*Pareto optimal*, *non-dominated*, *non-inferior*) solution of ($P^{*}$) if there exists no $x \in \mathbb {F}$ such that $F(x) \leqslant F(x^{\circ })$. In the area of multi-objective programming, there exist several versions of the notion of efficiency most of which are discussed in Miettinen (1999), Verma (2014), White (1982), Yu (1985). However, throughout this paper, we shall deal exclusively with the efficient solutions of (*P*) in the sense defined above.

For the purpose of comparison with the sufficient efficiency conditions that will be proposed and discussed in this paper, we next recall a set of necessary efficiency conditions for (*P*).

#### **Theorem 15**

(Zalmai 2013a) *Let*$x^{*}\in \mathbb {F}$, *let*$\lambda ^{*}=\varphi (x^{*})$, *for each*$i\in \underline{p}$, *let*$f_i$*and*$g_{i}$*be continuously differentiable at*$x^{*}$, *for each*$j\in \underline{q}$, *let the function*$G_j(\cdot ,t)$*be continuously differentiable at*$x^{*}$*for all*$t\in T_j$, *and for each*$k \in \underline{r}$, *let the function*$H_{k}(\cdot ,s)$*be continuously differentiable at*$x^{*}$*for all*$s \in S_{k}$. *If*$x^{*}$*is an efficient solution of (P), if the generalized Guignard constraint qualification holds at*$x^{*}$, *and if for each*$i_0\in \underline{p}$, *the set*$cone (\{\nabla G_j(x^{*}, t): t\in \hat{T}_j(x^{*}),j\in \underline{q}\}\cup \{\nabla _{i}(x^{*})-\lambda ^{*}_{i}\nabla g_{i}(x^{*}):i\in \underline{p}, i\ne i_0\})+ span \left( \{\nabla H_k(x^{*}, s): s\in S_k, k \in \underline{r}\}\right)$*is closed, then there exist*$u^{*} \in U$*and integers*$\nu _{0}^{*}$*and*$\nu ^{*}$, *with*$0 \leqq \nu _{0}^{*} \leqq \nu ^{*} \leqq n+1$, *such that there exist*$\nu _{0}^{*}$*indices*$j_{m}$, *with*$1 \leqq j_{m} \leqq q$, *together with*$\nu _{0}^{*}$*points*$t^{m} \in \hat{T}_{j_{m}}(x^{*}),\; m \in \underline{\nu _{0}^{*} },\; \nu ^{*}-\nu _{0}^{*}$*indices*$k_{m}$, *with*$1 \leqq k_{m} \leqq r$, *together with*$\nu ^{*}-\nu _{0}^{*}$*points*$s^{m}\in S_{k_{m}}$*for*$m \in \underline{\nu ^{*} }\backslash \underline{\nu _{0}^{*} }$, *and*$\nu ^{*}$*real numbers*$v^{*}_{m}$, *with*$v^{*}_{m} > 0$*for*$m \in \underline{\nu _{0}^{*} }$, *with the property that*

39 $$\begin{aligned} \sum _{i=1}^{p}u^{*}_{i} \left[ \nabla f_{i}(x^{*}) - \lambda ^{*}_{i}\nabla g_{i}(x^{*})\right] +\sum _{m=1}^{\nu _{0}^{*} }v^{*}_{m}\nabla G_{j_{m}}(x^{*},t^{m}) +\sum _{m=\nu _{0}^{*} +1}^{\nu ^{*} }v^{*}_{m}\nabla H_{k_{m}}(x^{*},s^{m}) = 0, \end{aligned}$$

*wherecone*(*V*) *is the conic hull of the set*$$V\subset \mathbb {R}^{n}$$*(i.e., the smallest convex cone containingV*), *span*(*V*) *is the linear hull ofV(i.e., the smallest subspace containingV)*, $$\hat{T}_{j}(x^{*})=\{t \in T_{j}:G_{j}(x^{*},t)=0\},$$$$U=\{u\in \mathbb {R}^{p}:u>0,\sum _{i=1}^{p}u_{i}=1\}$$, *and*$$\underline{\nu ^{*} }\backslash \underline{\nu _{0}^{*} }$$*is the complement of the set*$$\underline{\nu _{0}^{*} }$$*relative to the set*$$\underline{\nu ^{*} }$$.

### Random cases

Let the function $h(\cdot , z, \omega )$

40 $$\begin{aligned} h(x,z,\omega )=\left\langle \nabla f(x) + \frac{1}{4}\nabla ^2 f(x)z, z\right\rangle , \end{aligned}$$

be defined on the probability space $(\Omega ,\mathcal {F},P)$ where $\omega \in \Omega$ and *z* is a direction. We start with the assumptions we will use throughout the paper.

#### **Assumption 16**

1. $h(\cdot , \cdot , \omega )$ is an i.i.d. sequence (identically and independently distributed sequence).
2. $$\mathbb {E}[h(\cdot , \cdot , \omega )]<\infty$$.

Notice that (A1) implies that $\nabla _z h(\cdot , \cdot , \omega )$ is i.i.d. and is a function of $\omega$ and random variable. In the view of (A2), $\mathbb {E}[\nabla _z h(\cdot , \cdot , \omega )]<\infty .$

The following are the new definitions related with randomness which will be used for the main results.

#### **Definition 17**

Let *f* be a differentiable real-valued function defined on $\mathbb {R}^n$. Then *f* is said to be random $\eta$-asymptotic invex (invex with respect to $\eta$) at *y* if there exists a function $\eta : \mathbb {R}^n \times \Omega ^n \rightarrow \mathbb {R}^{n}$ such that for each $\omega \in \Omega ^n$,

41 $$\begin{aligned} f(\omega ) - f(y) \geqq \langle \nabla f(y),\mathbb {E}[\eta (\omega ,y)] \rangle , \end{aligned}$$

where $\nabla f(y) = (\partial f(y)/\partial y_{1}, \partial f(y)/\partial y_{2}, \ldots , \partial f(y)/\partial y_{n})$ is the gradient of *f* at *y*, and $\langle a,b \rangle$ denotes the inner product of the vectors *a* and *b*.

#### **Definition 18**

The function *F* is said to be random$(\alpha ,\eta )$*-V-asymptotic-invex at*$x^{*}$ if there exist functions $\alpha _{i} : \mathbb {R}^n \times \mathbb {R}^n \rightarrow \mathbb {R}_{+}\backslash \{0\}\equiv (0,\infty ),\; i \in \underline{N}$, and $\eta : \mathbb {R}^n \times \Omega ^n \rightarrow \mathbb {R}^{n}$ such that for each $\omega \in \Omega ^n$ and $i \in \underline{N}$,

42 $$\begin{aligned} F_{i}(x) - F_{i}(x^{*}) \geqq \langle \alpha _{i}(x,x^{*})\nabla F_{i}(x^{*}),\mathbb {E}[\eta (\omega ,x^{*})])\rangle . \end{aligned}$$

#### **Definition 19**

The function *F* is said to be random $(\beta ,\eta )$*-V-asymptotic-pseudoinvex at*$x^{*}$ if there exist functions $\beta _{i} : \mathbb {R}^n \times \mathbb {R}^n \rightarrow \mathbb {R}_{+}\backslash \{0\}, \; i \in \underline{N}$, and $\eta : \mathbb {R}^n \times \Omega ^n \rightarrow \mathbb {R}^{n}$ such that for each $\omega \in \Omega ^n$,

43 $$\begin{aligned} \left\langle \sum _{i=1}^{N}\nabla F_{i}(x^{*}), \mathbb {E}[\eta (\omega ,x^{*})])\right\rangle \geqq 0 \Rightarrow \sum _{i=1}^{N}\beta _{i}(x,x^{*})F_{i}(x) \geqq \sum _{i=1}^{N}\beta _{i}(x,x^{*})F_{i}(x^{*}). \end{aligned}$$

#### **Definition 20**

The function *F* is said to be random$(\gamma ,\eta )$*-V-asymptotic-quasiinvex at*$x^{*}$ if there exist functions $\gamma _{i} : \mathbb {R}^n \times \mathbb {R}^n \rightarrow \mathbb {R}_{+}\backslash \{0\}, \; i \in \underline{N}$, and $\eta : \mathbb {R}^n \times \Omega ^n \rightarrow \mathbb {R}^{n}$ such that for each $\omega \in \Omega ^n$,

44 $$\begin{aligned} \sum _{i=1}^{N}\gamma _{i}(x,x^{*})F_{i}(x) \leqq \sum _{i=1}^{N}\gamma _{i}(x,x^{*})F_{i}(x^{*}) \Rightarrow \left\langle \sum _{i=1}^{N}\nabla F_{i}(x^{*}), \mathbb {E}[\eta (\omega ,x^{*})])\right\rangle \leqq 0. \end{aligned}$$

Next, we define the exponential type Hanson–Antczak type generalized $HA(\alpha ,\beta ,\gamma ,\xi ,\eta ,\rho ,h(\cdot ,\cdot ,\omega ), \theta )$-V-asymptotic-invexities under the random function $h(\cdot ,\cdot ,\omega )$. Let the function $F = (F_{1},F_{2},\ldots ,F_{p}):X \rightarrow \mathbb {R}^{p}$ be differentiable at $x^{*}$.

#### **Definition 21**

The function *F* is said to be (strictly) $HA(\alpha ,\beta ,\gamma ,\xi ,\eta ,\rho ,h(\cdot ,\cdot ,\omega ),\theta )$-random V-asymptotic-invex at $x^{*}\in X$ if there exist functions $\alpha : X\times X \rightarrow \mathbb {R},\;\beta : X\times X \rightarrow \mathbb {R},\; \gamma _{i} : X\times X \rightarrow \mathbb {R}_{+}, \; \xi _{i} : X\times X \rightarrow \mathbb {R}_{+}\backslash \{0\},\; i \in \underline{p},\; z\in \mathbb {R}^{n}, \eta :X\times X\rightarrow \mathbb {R}^n\; \rho _{i} : X\times X \rightarrow \mathbb {R},\; i \in \underline{p}$, and $\theta : X\times X \rightarrow \mathbb {R}^{n}$ such that for all $x \in X \;(x \ne x^{*})$ and $i \in \underline{p}$,

45 $$\begin{aligned}&\frac{1}{\alpha (x,x^{*})}\gamma _{i}(x,x^{*})\left( e^{\alpha (x,x^{*})\left[ F_{i}(x) - F_{i}(x^{*})\right] } - 1\right) \nonumber \\&\quad ({>}) \geqq \frac{1}{\beta (x,x^{*})}\left\langle \xi _{i}(x,x^{*})\mathbb {E}\left[ \nabla _{z}h_i(x^{*},z,\omega )\right] ,e^{\beta (x,x^{*})\eta (x,x^*)} - \mathbf {1}\right\rangle \nonumber \\&\qquad + \rho _{i}(x,x^{*})\Vert \theta (x,x^{*})\Vert ^{2}\quad \text{ if } \alpha (x,x^{*})\ne 0 \text{ and } \beta (x,x^{*})\ne 0 \;\;\text{ for}\;\text{all}\; x \in X, \end{aligned}$$

46 $$\begin{aligned}&\frac{1}{\alpha (x,x^{*})}\gamma _{i}(x,x^{*})\left( e^{\alpha (x,x^{*})\left[ F_{i}(x) - F_{i}(x^{*})\right] } - 1\right) ({>}) \geqq \left\langle \xi _{i}(x,x^{*})\mathbb {E}\left[ \nabla _{z}h_i(x^{*},z,\omega )\right] ,\eta (x,x^*)\right\rangle \nonumber \\&\quad + \rho _{i}(x,x^{*})\Vert \theta (x,x^{*})\Vert ^{2} \quad \text{ if } \alpha (x,x^{*}) \ne 0 \text{ and } \beta (x,x^{*})\rightarrow 0 \;\;\text{ for}\;\text{all}\; x \in X, \end{aligned}$$

47 $$\begin{aligned}&\gamma _{i}(x,x^{*})\left[ F_{i}(x) - F_{i}(x^{*})\right] ({>}) \geqq \frac{1}{\beta (x,x^{*})}\left\langle \xi _{i}(x,x^{*})\mathbb {E}\left[ \nabla _{z}h_i(x^{*},z,\omega )\right] ,e^{\beta (x,x^{*})\eta (x,x^*)} - \mathbf {1}\right\rangle \nonumber \\&\quad +\rho _{i}(x,x^{*})\Vert \theta (x,x^{*})\Vert ^{2} \quad \text{ if } \alpha (x,x^{*})\rightarrow 0 \text{ and } \beta (x,x^{*}) \ne 0 \;\;\text{ for}\;\text{all}\; x \in X, \end{aligned}$$

48 $$\begin{aligned}&\gamma _{i}(x,x^{*})\left[ F_{i}(x) - F_{i}(x^{*})\right] ({>}) \geqq \left\langle \xi _{i}(x,x^{*})\mathbb {E}\left[ \nabla _{z}h_i(x^{*},z,\omega )\right] ,\eta (x,x^*) \right\rangle + \rho _{i}(x,x^{*})\Vert \theta (x,x^{*})\Vert ^{2} \nonumber \\&\quad \text{ if } \alpha (x,x^{*})\rightarrow 0 \text{ and } \beta (x,x^{*}) \rightarrow 0 \;\;\text{ for}\;\text{all}\; x \in X, \end{aligned}$$

where $\Vert \cdot \Vert$ is a norm on $\mathbb {R}^{n}$ and

49 $$\begin{aligned} \left( e^{\beta (x,x^{*})\eta _(x,x^*)} - \mathbf {1}\right) \equiv \left( e^{\beta (x,x^{*})\eta _{1}(x,x^*)} - 1,\ldots ,e^{\beta (x,x^{*})\eta _{n}(x,x^*)} - 1\right) , \end{aligned}$$

with $h:\mathbb {R}^n\times \mathbb {R}^n\times \Omega ^{n} \rightarrow \mathbb {R}^n$ differentiable and random function. The function *F* is said to be (strictly) $HA(\alpha ,\beta ,\gamma ,\xi ,\eta ,\rho ,h(\cdot ,\cdot ,\omega ),\theta )$-random V-invex $x^{*}\in X$ if the expectation is dropped in the above definition.

#### **Definition 22**

The function *F* is said to be (strictly) $HA(\alpha ,\beta ,\gamma ,\xi ,\eta ,\rho ,h(\cdot ,\cdot ,\omega ),\theta )$-random V-asymptotic-pseudoinvex at $x^{*}\in X$ if there exist functions $\alpha : X\times X \rightarrow \mathbb {R}, \; \beta : X\times X \rightarrow \mathbb {R}, \; \gamma : X\times X \rightarrow \mathbb {R}_{+}, \; \xi _{i} : X\times X \rightarrow \mathbb {R}_{+}\backslash \{0\}, \; i \in \underline{p}, \; z \in \mathbb {R}^{n}, \;\eta :X\times X\rightarrow \mathbb {R}^n$, $\rho : X\times X \rightarrow \mathbb {R}$, and $\theta : X\times X \rightarrow \mathbb {R}^{n}$ such that for all $x \in X \;(x \ne x^{*})$,

50 $$\begin{aligned}&\frac{1}{\beta (x,x^{*})}\left\langle \sum _{i=1}^{p}\mathbb {E}\left[ \nabla _z h_i(x^*,z,\omega )\right] ,e^{\beta (x,x^{*})\eta (x,x^*))} - \mathbf {1}\right\rangle \geqq - \rho (x,x^{*})\Vert \theta (x,x^{*})\Vert ^{2} \nonumber \\&\quad \Rightarrow \frac{1}{\alpha (x,x^{*})}\gamma (x,x^{*})\left( e^{\alpha (x,x^{*})\sum _{i=1}^{p}\xi _{i}(x,x^{*})\left[ F_{i}(x) - F_{i}(x^{*})\right] } - 1\right) ({>}) \geqq 0 \nonumber \\&\qquad \text{ if } \alpha (x,x^{*})\ne 0 \text{ and } \beta (x,x^{*})\ne 0 \;\;\text{ for}\;\text{all}\; x \in X, \end{aligned}$$

51 $$\begin{aligned}&\left\langle \sum _{i=1}^{p}\mathbb {E}\left[ \nabla _z h_{i}(x^{*},z,\omega )\right] ,\eta (x,x^{*})\right\rangle \geqq - \rho (x,x^{*})\Vert \theta (x,x^{*})\Vert ^{2} \nonumber \\&\quad \Rightarrow \frac{1}{\alpha (x,x^{*})}\gamma (x,x^{*})\left( e^{\alpha (x,x^{*})\sum _{i=1}^{p}\xi _{i}(x,x^{*})\left[ F_{i}(x) - F_{i}(x^{*})\right] } - 1\right) ({>}) \geqq 0 \nonumber \\&\qquad \text{ if } \alpha (x,x^{*}) \ne 0 \text{ and } \beta (x,x^{*}) \rightarrow 0 \;\;\text{ for}\;\text{all}\; x \in X, \end{aligned}$$

52 $$\begin{aligned}&\frac{1}{\beta (x,x^{*})}\left\langle \sum _{i=1}^{p}\mathbb {E}\left[ \nabla _z h_{i}(x^{*},z,\omega )\right] ,e^{\beta (x,x^{*})\eta (x,x^{*})} - \mathbf {1}\right\rangle \geqq - \rho (x,x^{*})\Vert \theta (x,x^{*})\Vert ^{2} \nonumber \\&\quad \Rightarrow \gamma (x,x^{*})\sum _{i=1}^{p}\xi _{i}(x,x^{*})\left[ F_{i}(x) - F_{i}(x^{*})\right] ({>}) \geqq 0 \nonumber \\&\qquad \text{ if } \alpha (x,x^{*}) \rightarrow 0 \text{ and } \beta (x,x^{*}) \ne 0 \;\;\text{ for}\;\text{all}\; x \in X, \end{aligned}$$

53 $$\begin{aligned}&\left\langle \sum _{i=1}^{p}\mathbb {E}\left[ \nabla _z h_{i}(x^{*},z,\omega )\right] ,\eta (x,x^{*})\right\rangle \geqq - \rho (x,x^{*})\Vert \theta (x,x^{*})\Vert ^{2} \nonumber \\&\quad \Rightarrow \gamma (x,x^{*})\sum _{i=1}^{p}\xi _{i}(x,x^{*})\left[ F_{i}(x) - F_{i}(x^{*})\right] ({>}) \geqq 0 \nonumber \\&\qquad \text{ if } \alpha (x,x^{*}) \rightarrow 0 \text{ and } \beta (x,x^{*}) \rightarrow 0 \;\;\text{ for}\;\text{all}\; x \in X. \end{aligned}$$

with $h:\mathbb {R}^n\times \mathbb {R}^n\times \Omega ^{n} \rightarrow \mathbb {R}^n$ differentiable and random function. The function *F* is said to be (strictly) $HA(\alpha ,\beta ,\gamma ,\xi ,\eta ,\rho ,h(\cdot ,\cdot ,\omega ),\theta )$-random V-pseudoinvex $x^{*}\in X$ if the expectation is dropped in the above definition.

#### **Definition 23**

The function *F* is said to be (prestrictly) $HA(\alpha ,\beta ,\gamma ,\xi ,\eta ,\rho ,h(\cdot ,\cdot ,\omega ),\theta )$-random V-asymptotic-quasiinvex at $x^{*}\in X$ if there exist functions $\alpha : X\times X \rightarrow \mathbb {R}, \; \beta : X\times X \rightarrow \mathbb {R},\; \gamma : X\times X \rightarrow \mathbb {R}_{+},\; \xi _{i} : X\times X \rightarrow \mathbb {R}_{+}\backslash \{0\}, \; i \in \underline{p},\; \eta : X\times X \rightarrow \mathbb {R}^{n},\; \rho : X\times X \rightarrow \mathbb {R}$, and $\theta : X\times X \rightarrow \mathbb {R}^{n}$ such that for all $x \in X$,

54 $$\begin{aligned}&\frac{1}{\alpha (x,x^{*})}\gamma (x,x^{*})\left( e^{\alpha (x,x^{*})\sum _{i=1}^{p}\xi _{i}(x,x^{*})\left[ F_{i}(x) - F_{i}(x^{*})\right] } - 1\right) ({<}) \leqq 0 \nonumber \\&\quad \Rightarrow \frac{1}{\beta (x,x^{*})}\left\langle \sum _{i=1}^{p}\mathbb {E}\left[ \nabla _z h_{i}(x^{*},z,\omega )\right] ,e^{\beta (x,x^{*})\eta (x,x^{*})} - \mathbf {1}\right\rangle \leqq - \rho (x,x^{*})\Vert \theta (x,x^{*})\Vert ^{2} \nonumber \\&\qquad \text{ if } \alpha (x,x^{*})\ne 0 \text{ and } \beta (x,x^{*})\ne 0 \;\;\text{ for}\;\text{all}\; x \in X, \end{aligned}$$

55 $$\begin{aligned}&\frac{1}{\alpha (x,x^{*})}\gamma (x,x^{*})\left( e^{\alpha (x,x^{*})\sum _{i=1}^{p}\xi _{i}(x,x^{*})\left[ F_{i}(x) - F_{i}(x^{*})\right] } - 1\right) ({<}) \leqq 0 \nonumber \\&\quad \Rightarrow \left\langle \sum _{i=1}^{p}\mathbb {E}\left[ \nabla _z h_{i}(x^{*},z,\omega )\right] ,\eta (x,x^{*})\right\rangle \leqq - \rho (x,x^{*})\Vert \theta (x,x^{*})\Vert ^{2} \nonumber \\&\qquad \text{ if } \alpha (x,x^{*}) \ne 0 \text{ and } \beta (x,x^{*}) \rightarrow 0 \;\;\text{ for}\;\text{all}\; x \in X, \end{aligned}$$

56 $$\begin{aligned}&\gamma (x,x^{*})\sum _{i=1}^{p}\xi _{i}(x,x^{*})\left[ F_{i}(x) - F_{i}(x^{*})\right] ({<}) \leqq 0 \nonumber \\&\quad \Rightarrow \frac{1}{\beta (x,x^{*})}\left\langle \sum _{i=1}^{p}\mathbb {E}\left[ \nabla _z h_{i}(x^{*},z,\omega )\right] ,e^{\beta (x,x^{*})\eta (x,x^{*})} - \mathbf {1}\right\rangle \leqq - \rho (x,x^{*})\Vert \theta (x,x^{*})\Vert ^{2} \nonumber \\&\qquad \text{ if } \alpha (x,x^{*}) \rightarrow 0 \text{ and } \beta (x,x^{*}) \ne 0 \;\;\text{ for}\;\text{all}\; x \in X, \end{aligned}$$

57 $$\begin{aligned}&\gamma (x,x^{*})\sum _{i=1}^{p}\xi _{i}(x,x^{*})\left[ F_{i}(x) - F_{i}(x^{*})\right] ({<}) \leqq 0 \nonumber \\&\quad \Rightarrow\left\langle \sum _{i=1}^{p}\mathbb {E}\left[ \nabla _z h_{i}(x^{*},z,\omega )\right] ,\eta (x,x^{*}) \right\rangle \leqq - \rho (x,x^{*})\Vert \theta (x,x^{*})\Vert ^{2} \nonumber \\&\qquad \text{ if } \alpha (x,x^{*}) \rightarrow 0 \text{ and } \beta (x,x^{*}) \rightarrow 0 \;\;\text{ for}\;\text{all}\; x \in X. \end{aligned}$$

with $h:\mathbb {R}^n\times \mathbb {R}^n\times \Omega ^{n} \rightarrow \mathbb {R}^n$ differentiable and random function. The function *F* is said to be (strictly) $HA(\alpha ,\beta ,\gamma ,\xi ,\eta ,\rho ,h(\cdot ,\cdot ,\omega ),\theta )$-random V-quasiinvex $x^{*}\in X$ if the expectation is dropped in the above definition.

## Asymptotic sufficiency conditions

In this section, we present several sets of asymptotic sufficiency results in which various generalized exponential type $HA(\alpha ,\beta ,\gamma ,\xi ,\eta ,\rho ,h(\cdot ,\cdot ,\cdot ),\theta )$-V-invexity assumptions are imposed on certain vector functions whose components are the individual as well as some combinations of the problem functions.

Let the function $\mathcal {E}_{i}(\cdot ,\lambda ,u) : X \rightarrow \mathbb {R}$ be defined, for fixed $\lambda$ and *u*, on *X* by

58 $$\begin{aligned} \mathcal {E}_{i}(z,\lambda ,u) = u_{i}[f_{i}(z) - \lambda _i g_{i}(z)], \quad i \in \underline{p}. \end{aligned}$$

### **Theorem 24**

*Let*$x^{*} \in \mathbb {F}$, *let*$\lambda ^{*} = \varphi (x^{*})$, *let the functions*$f_{i}, \, g_{i},\; i \in \underline{p},\; G_{j}(\cdot ,t)$, *and*$H_{k}(\cdot ,s)$*be differentiable at*$x^{*}$*for all*$t \in T_{j}$*and*$s \in S_{k},\; j \in \underline{q},\; k \in \underline{r}$, *and assume that Assumption* 16*is satisfied and that there exist*$u^{*} \in U$*and integers*$\nu _{0}$*and*$\nu$, *with*$0 \leqq \nu _{0} \leqq \nu \leqq n+1$, *such that there exist*$\nu _{0}$*indices*$j_{m}$, *with*$1 \leqq j_{m} \leqq q$, *together with*$\nu _{0}$*points*$t^{m} \in \hat{T}_{j_{m}}(x^{*}),\; m \in \underline{\nu _{0}},\;\nu - \nu _{0}$*indices*$k_{m}$, *with*$1 \leqq k_{m} \leqq r$, *together with*$\nu - \nu _{0}$*points*$s^{m}\in S_{k_{m}}, \; m \in \underline{\nu }\backslash \underline{\nu _{0}}$, *and*$\nu$*real numbers*$v^{*}_{m}$*with*$v^{*}_{m} > 0$*for*$m\in \underline{\nu _{0}}$, *with*$d=\min \{u_{i}^*,v_{m}^*\}$, *and with the property that*

59 $$\begin{aligned}&d\cdot \left[ \mathbb {E}\left[ \nabla _z h_{i}(x^{*},z,\omega )\right] -\bar{\lambda }\mathbb {E}\left[ \nabla _z \kappa _{i}(x^{*},z,\omega )\right] \right] +d\cdot \mathbb {E}\left[ \nabla _z \psi _{j_{m}}(x^{*},t^{m},z,\omega )\right] \nonumber \\&\quad +d\cdot \mathbb {E}\left[ \nabla _z \varpi _{k_{m}}(x^{*},s^{m},z,\omega )\right] =0. \end{aligned}$$

*or*

60 $$\begin{aligned}&\sum _{i=1}^{p}u^{*}_{i}\left[ \mathbb {E}\left[ \nabla _z h_{i}(x^{*},z,\omega )\right] - \lambda _i^{*} \mathbb {E}\left[ \nabla _z \kappa _{i}(x^{*},z,\omega )\right] \right] + \sum _{m=1}^{\nu _{0}}v^{*}_{m}\mathbb {E}\left[ \nabla _z \psi _{j_{m}}(x^{*},t^{m},z,\omega )\right] \nonumber \\&\quad +\sum _{m=\nu _{0}+1}^{\nu }v^{*}_{m}\mathbb {E}\left[ \nabla _z \varpi _{k_{m}}(x^{*},s^{m},z,\omega )\right] = 0. \end{aligned}$$

*Assume, furthermore, that either one of the following three sets of conditions holds under the random function h:*

(a)
  (i): $f_{i}$*is exponential type*$HA(\alpha ,\beta ,\bar{\gamma },\xi ,\eta ,\bar{\rho },h(\cdot ,\cdot ,\omega ),\theta )$-*random V-invex* at $x^{*}$, $g_{i}$*is exponential type*$HA(\alpha ,\beta ,\bar{\gamma },\xi ,\eta ,\bar{\rho },\kappa (\cdot ,\cdot ,\omega ),\theta )$-*random V-invex at*$x^{*}$, *and*$\bar{\gamma }(x,x^{*}) > 0$*for all*$x \in \mathbb {F}$;
  (ii): $(v^{*}_{1}G_{j_{1}}(\cdot ,t^{1}),\ldots ,v^{*}_{\nu _{0}}G_{j_{\nu _{0}}}(\cdot ,t^{\nu _{0}}))$*is exponential type*$HA(\alpha ,\beta ,\hat{\gamma },\pi ,\eta ,\hat{\rho },\psi (\cdot ,\cdot ,\omega ),\theta )$-*random V-invex at*$x^{*}$;
  (iii): $(v^{*}_{\nu _{0}+1}H_{k_{\nu _{0}+1}}(\cdot ,s^{\nu _{0}+1}),\dots ,v^{*}_{\nu }H_{k_{\nu }}(\cdot ,s^{\nu }))$*is exponential type*$HA(\alpha ,\beta ,\breve{\gamma },\delta ,\eta ,\breve{\rho },\varpi (\cdot ,\cdot ,\omega ),\theta )$*-random V-invex at*$x^{*}$;
  (iv): $\xi _{i} = \pi _k=\delta _l=\sigma$*for all*$i \in \underline{p},\; k\in \underline{\nu _0},$ and $l \in \underline{\nu }\backslash \underline{\nu _0}$;
  (v): $$\sum _{i=1}^{p}u^{*}_{i}\bar{\rho }_{i}(x,x^{*}) +\sum _{m=1}^{\nu _0}\hat{\rho }_m(x,x^{*}) + \sum _{m=\nu _0+1}^{\nu } \breve{\rho }_m(x,x^{*}) \geqq 0$$ for all $$x \in \mathbb {F}$$;
(b)
  (i): $f_{i}$*is exponential type*$HA(\alpha ,\beta ,\bar{\gamma },\xi ,\eta ,\bar{\rho },h(\cdot ,\cdot ,\omega ),\theta )$-*random V-asymptotic-invex at*$x^{*}$, $g_{i}$*is exponential type*$HA(\alpha ,\beta ,\bar{\gamma },\xi ,\eta ,\bar{\rho },\kappa (\cdot ,\cdot ,\omega ),\theta )$-*random V-asymptotic-invex at*$x^{*}$, and $\bar{\gamma }(x,x^{*}) > 0$*for all*$x \in \mathbb {F}$;
  (ii): $(v^{*}_{1}G_{j_{1}}(\cdot ,t^{1}),\ldots ,v^{*}_{\nu _{0}}G_{j_{\nu _{0}}}(\cdot ,t^{\nu _{0}}))$*is exponential type*$HA(\alpha ,\beta ,\hat{\gamma },\pi ,\eta ,\hat{\rho },\psi (\cdot ,\cdot ,\omega ),\theta )$-*random V-asymptotic-invex at*$x^{*}$;
  (iii): $(v^{*}_{\nu _{0}+1}H_{k_{\nu _{0}+1}}(\cdot ,s^{\nu _{0}+1}),\dots ,v^{*}_{\nu }H_{k_{\nu }}(\cdot ,s^{\nu }))$*is exponential type*$HA(\alpha ,\beta ,\breve{\gamma },\delta ,\eta ,\breve{\rho },\varpi (\cdot ,\cdot ,\omega ),\theta )$*-random V-asymptotic-invex at*$x^{*}$;
  (iv): $\xi _{i} = \pi _k=\delta _l=\sigma$*for all*$i \in \underline{p},\; k\in \underline{\nu _0},$*and*$l \in \underline{\nu }\backslash \underline{\nu _0}$;
  (v): $$\sum _{i=1}^{p}u^{*}_{i}\bar{\rho }_{i}(x,x^{*}) +\sum _{m=1}^{\nu _0}\hat{\rho }_m(x,x^{*}) + \sum _{m=\nu _0+1}^{\nu } \breve{\rho }_m(x,x^{*}) \geqq 0$$ for all $$x \in \mathbb {F}$$;
(c): *the function*$(L_{1}(\cdot , u^{*},v^{*},\lambda ^{*} ,\bar{t},\bar{s}),\ldots ,L_{p}(\cdot ,u^{*},v^{*},\lambda ^{*},\bar{t},\bar{s}))$*is exponential type*$HA(\alpha ,\beta ,\gamma ,\xi ,\eta ,\rho ,h(\cdot ,\cdot ,\omega ),\kappa (\cdot ,\cdot ,\omega ),\psi (\cdot ,\cdot ,\omega ),\varpi (\cdot ,\cdot ,\omega ),\theta )$*-random V-asymptotic-pseudoinvex at*$x^{*}$*and*$\gamma (x,x^{*}) > 0$*for all*$x \in \mathbb {F}$, *where*
  61 $$\begin{aligned} &L_{i}(z,u^{*},v^{*},\lambda ^{*},\bar{t},\bar{s})\\ &\quad=u^{*}_{i}\left[ f_{i}(z)-\lambda _i^{*}g_{i}(z)+ \sum _{m=1}^{\nu _{0}}v^{*}_{m}G_{j_{m}}(z,t^{m})+\sum _{m=\nu _{0}+1}^{\nu }v^{*}_{m}H_{k_{m}}(z,s^{m})\right] ,\;\;\; i \in \underline{p}. \end{aligned}$$

*Then*$x^{*}$*is an efficient solution of (P)*.

### *Proof*

(a) In view of our assumptions in (i)–(iv), we have

62 $$\begin{aligned}&\frac{1}{\alpha (x,x^{*})}\bar{\gamma }_{i}(x,x^{*})\left( e^{\alpha (x,x^{*})\{f_{i}(x) - \lambda ^{*}_{i}g_{i}(x) - [f_{i}(x^{*}) - \lambda ^{*}_{i}g_{i}(x^{*})]\}} - 1\right) \nonumber \\&\quad \geqq \frac{1}{\beta (x,x^{*})}\left\langle \sigma (x,x^{*})\left[ \mathbb {E}\left[ \nabla _z h_{i}(x^{*},z,\omega )\right] - \lambda ^{*}_{i}\mathbb {E}\left[ \nabla _z \kappa _{i}(x^{*},z,\omega )\right] \right] ,e^{\beta (x,x^{*})\eta (x,x^*)} - \mathbf {1}\right\rangle \nonumber \\&\qquad +\bar{\rho }_{i}(x,x^{*})\Vert \theta (x,x^{*})\Vert ^{2},\quad i \in \underline{p}, \end{aligned}$$

63 $$\begin{aligned}&\frac{1}{\alpha (x,x^{*})}\hat{\gamma }_{m}(x,x^{*})\left( e^{\alpha (x,x^{*})[v^{*}_{m}G_{j_{m}}(x,t^{m}) - v^{*}_{m}G_{j_{m}}(x^{*},t^{m})]} - 1\right) \nonumber \\&\quad \geqq \frac{1}{\beta (x,x^{*})}\left\langle \sigma (x,x^{*})v^{*}_{m}\mathbb {E}\left[ \nabla _z \psi _{j_{m}}(x^{*},t^{m},z,\omega )\right] ,e^{\beta (x,x^{*})\eta (x,x^*)} - \mathbf {1}\right\rangle \nonumber \\&\qquad + \hat{\rho }_{m}(x,x^{*})\Vert \theta (x,x^{*})\Vert ^{2}, \quad m \in \underline{\nu _{0}}, \end{aligned}$$

64 $$\begin{aligned}&\frac{1}{\alpha (x,x^{*})}\breve{\gamma }_{m}(x,x^{*})\left( e^{\alpha (x,x^{*})[v^{*}_{m}H_{k_{m}}(x,s^{m}) - v^{*}_{m}H_{k_{m}}(x^{*},s^{m})]} - 1\right) \nonumber \\&\quad \geqq \frac{1}{\beta (x,x^{*})}\left\langle \sigma (x,x^{*})v^{*}_{m}\mathbb {E}\left[ \nabla _z \varpi _{k_{m}}(x^{*},s^{m},z,\omega )\right] ,e^{\beta (x,x^{*})\eta (x,x^*)} - \mathbf {1}\right\rangle \nonumber \\&\qquad + \breve{\rho }_{m}(x,x^{*})\Vert \theta (x,x^{*})\Vert ^{2}, \quad m \in \underline{\nu }\backslash \underline{\nu _{0}}. \end{aligned}$$

Multiplying (61) by $u^{*}_{i}$ and then summing over $i \in \underline{p}$, summing (62) over $m \in \underline{\nu _{0}}$, and summing (63) over $m \in \underline{\nu }\backslash \underline{\nu _{0}}$, and finally adding the resulting inequalities, we get

65 $$\begin{aligned}&\frac{1}{\alpha (x,x^{*})}\left\{ \sum _{i=1}^{p}u^{*}_{i}\bar{\gamma }_{i}(x,x^{*})\left( e^{\alpha (x,x^{*})\{f_{i}(x) - \lambda ^{*}_{i}g_{i}(x)-[f_{i}(x^{*})-\lambda ^{*}_{i}g_{i}(x^{*})]\}}-1\right) \right. \nonumber \\&\quad \left. +\sum _{m=1}^{\nu _{0}}\hat{\gamma }_{m}(x,x^{*})\left( e^{\alpha (x,x^{*})[v^{*}_{m}G_{j_{m}}(x,t^{m})-v^{*}_{m}G_{j_{m}}(x^{*},t^{m})]} -1\right) \right. \nonumber \\&\quad \left. +\sum _{m=\nu _{0}+1}^{\nu }\breve{\gamma }_{m}(x,x^{*})\left( e^{\alpha (x,x^{*})[v^{*}_{m}H_{k_{m}}(x,s^{m})-v^{*}_{m}H_{k_{m}}(x^{*},s^{m})]} - 1\right) \right\} \nonumber \\&\quad \geqq \frac{1}{\beta (x,x^{*})}\sigma (x,x^{*})\left\langle \sum _{i=1}^{p}u^{*}_{i}\left[ \mathbb {E}\left[ \nabla _z h_{i}(x^{*},z,\omega )\right] - \lambda ^{*}_{i}\mathbb {E}\left[ \nabla _z \kappa _{i}(x^{*},z,\omega )\right] \right]\right.\\&\qquad +\sum _{m=1}^{\nu _{0}}v^{*}_{m}\mathbb {E}\left[ \nabla _z \psi _{j_{m}}(x^{*},t^{m},z,\omega )\right] \nonumber \\&\qquad \left. +\sum _{m=\nu _{0} + 1}^{\nu }v^{*}_{m}\mathbb {E}\left[ \nabla _z \varpi _{k_{m}}(x^{*},s^{m},z,\omega )\right] ,e^{\beta (x,x^{*})\eta (x,x^*)}- \mathbf {1}\right\rangle \nonumber \\&\qquad +\left[ \sum _{i=1}^{p}u^{*}_{i}\bar{\rho }_{i}(x,x^{*}) + \sum _{m=1}^{\nu _{0}}\hat{\rho }_{m}(x,x^{*}) + \sum {m =\nu _{0}+1}^{\nu }\breve{\rho }_{m}(x,x^{*})\right] \Vert \theta (x,x^{*})\Vert ^{2}. \end{aligned}$$

Let $\ell =\min \{p,\nu \}.$ Then the last term of above inequality

66 $$\begin{aligned}&\frac{1}{\beta (x,x^{*})}\sigma (x,x^{*})\left\langle \sum _{i=1}^{p}u^{*}_{i}\left[ \mathbb {E}\left[ \nabla _z h_{i}(x^{*},z,\omega )\right] -\lambda ^{*}_{i}\mathbb {E}\left[ \nabla _z \kappa _{i}(x^{*},z,\omega )\right] \right]\right.\\&\quad+\sum _{m=1}^{\nu _{0}}v^{*}_{m}\mathbb {E}\left[ \nabla _z \psi _{j_{m}}(x^{*},t^{m},z,\omega )\right] \nonumber \\&\quad \left. +\sum _{m=\nu _{0}+1}^{\nu }v^{*}_{m}\mathbb {E}\left[ \nabla _z \varpi _{k_{m}}(x^{*},s^{m},z,\omega )\right] ,e^{\beta (x,x^{*}) \eta (x,x^*)}-\mathbf {1}\right\rangle \nonumber \\&\quad +\left[\sum _{i=1}^{p}u^{*}_{i}\bar{\rho }_{i}(x,x^{*}) +\sum _{m=1}^{\nu_{0}}\hat{\rho }_{m}(x,x^{*})+\sum _{m =\nu _{0}+1}^{\nu}\breve{\rho }_{m}(x,x^{*})\right] \Vert \theta (x,x^{*})\Vert^{2}\nonumber \\&\quad \geqq \frac{1}{\beta (x,x^{*})}\sigma(x,x^{*})\left\langle \frac{1}{\ell }\sum _{i=1}^{p}u^{*}_{i}\left[\mathbb {E}\left[ \nabla _z h_{i}(x^{*},z,\omega )\right] - \lambda^{*}_{i}\mathbb {E}\left[ \nabla _z \kappa _{i}(x^{*},z,\omega)\right] \right]\right.\\&\qquad +\frac{1}{\ell } \sum _{m=1}^{\nu _{0}}v^{*}_{m} \mathbb{E}\left[ \nabla _z \psi _{j_{m}}(x^{*},t^{m},z,\omega )\right] \nonumber \\&\qquad \left. +\frac{1}{\ell } \sum _{m=\nu_{0} + 1}^{\nu }v^{*}_{m} \mathbb {E}\left[ \nabla _z \varpi_{k_{m}}(x^{*},s^{m},z,\omega )\right] , e^{\beta (x,x^{*})\eta(x,x^*)}- \mathbf {1}\right\rangle \nonumber \\&\qquad +\left[ \sum _{i=1}^{p}u^{*}_{i} \bar{\rho}_{i}(x,x^{*}) + \sum _{m=1}^{\nu _{0}} \hat{\rho}_{m}(x,x^{*})+\sum _{m =\nu _{0}+1}^{\nu } \breve{\rho}_{m}(x,x^{*})\right] \Vert \theta (x,x^{*})\Vert ^{2}.\end{aligned}$$

Let $d=\min \{u^{*}_{i},v^{*}_{m}\}$ and by using the law of large number, we get

67 $$\begin{aligned}&\frac{1}{\beta (x,x^{*})}\sigma (x,x^{*})\left\langle \sum _{i=1}^{p}u^{*}_{i}\left[ \mathbb {E}\left[ \nabla _z h_{i}(x^{*},z,\omega )\right] - \lambda ^{*}_{i}\mathbb {E}\left[ \nabla _z \kappa _{i}(x^{*},z,\omega )\right] \right] +\sum _{m=1}^{\nu _{0}}v^{*}_{m}\mathbb {E}\left[ \nabla _z \psi _{j_{m}}(x^{*},t^{m},z,\omega )\right] \right. \nonumber \\&\quad \left. +\sum _{m=\nu _{0} + 1}^{\nu }v^{*}_{m}\mathbb {E}\left[ \nabla _z \varpi _{k_{m}}(x^{*},s^{m},z,\omega )\right] ,e^{\beta (x,x^{*})\eta (x,x^*)}-\mathbf {1}\right\rangle \nonumber \\&\quad +\left[ \sum _{i=1}^{p}u^{*}_{i}\bar{\rho }_{i}(x,x^{*})+\sum _{m=1}^{\nu _{0}}\hat{\rho }_{m}(x,x^{*})+\sum _{m =\nu _{0}+1}^{\nu }\breve{\rho }_{m}(x,x^{*})\right] \Vert \theta (x,x^{*})\Vert ^{2}\nonumber \\&\quad \geqq \frac{1}{\beta (x,x^{*})}\sigma (x,x^{*})\left\langle \frac{1}{\ell }\sum _{i=1}^{p}u^{*}_{i}\left[ \mathbb {E}\left[ \nabla _z h_{i}(x^{*},z,\omega )\right] - \lambda ^{*}_{i}\mathbb {E}\left[ \nabla _z \kappa _{i}(x^{*},z,\omega )\right] \right] +\frac{1}{\ell } \sum _{m=1}^{\nu _{0}}v^{*}_{m}\mathbb {E}\left[ \nabla _z \psi _{j_{m}}(x^{*},t^{m},z,\omega )\right] \right. \nonumber \\&\qquad \left. +\frac{1}{\ell } \sum _{m=\nu _{0} + 1}^{\nu }v^{*}_{m}\mathbb {E}\left[ \nabla _z \varpi _{k_{m}}(x^{*},s^{m},z,\omega )\right] ,e^{\beta (x,x^{*})\eta (x,x^*)} - \mathbf {1}\right\rangle \nonumber \\&\qquad +\left[ \sum _{i=1}^{p}u^{*}_{i}\bar{\rho }_{i}(x,x^{*}) + \sum _{m=1}^{\nu _{0}}\hat{\rho }_{m}(x,x^{*}) + \sum _{m =\nu _{0}+1}^{\nu }\breve{\rho }_{m}(x,x^{*})\right] \Vert \theta (x,x^{*})\Vert ^{2}\nonumber \\&\quad \geqq \frac{1}{\beta (x,x^{*})}\sigma (x,x^{*})\left\langle d\cdot \left[ \mathbb {E}\left[ \nabla _z h_{i}(x^{*},z,\omega )\right] - \bar{\lambda }\mathbb {E}\left[ \nabla _z \kappa _{i}(x^{*},z,\omega )\right] \right] +d\cdot \mathbb {E}\left[ \nabla _z \psi _{j_{m}}(x^{*},t^{m},z,\omega )\right] \right. \nonumber \\&\qquad \left. +d\cdot \mathbb {E}\left[ \nabla _z \varpi _{k_{m}}(x^{*},s^{m},z,\omega )\right] ,e^{\beta (x,x^{*})\eta (x,x^*)} -\mathbf {1}\right\rangle \nonumber \\&\qquad +\left[ \sum _{i=1}^{p}u^{*}_{i}\bar{\rho }_{i}(x,x^{*})+\sum _{m=1}^{\nu _{0}}\hat{\rho }_{m}(x,x^{*})+\sum _{m =\nu _{0}+1}^{\nu }\breve{\rho }_{m}(x,x^{*})\right] \Vert \theta (x,x^{*})\Vert ^{2}. \end{aligned}$$

Now using (59) and the condition (v), and noticing that $\sigma (x,x^{*}) > 0,\; \varphi (x^{*}) = \lambda ^{*}; \;x,\; x^{*} \in \mathbb {F}$, and $G_{j_{m}}(x^{*},t^{m}) = 0$ for all $m \in \underline{\nu _{0}}$, the above inequality reduces to

68 $$\begin{aligned} \frac{1}{\alpha (x,x^{*})}\sum _{i=1}^{p}u^{*}_{i}\bar{\gamma }_{i}(x,x^{*})\left( e^{\alpha (x,x^{*})[f_{i}(x) - \lambda ^{*}_{i}g_{i}(x)]} - 1\right) \geqq 0. \end{aligned}$$

Since $\gamma (x,x^*) > 0$, even if we consider the both cases $\alpha (x,x^*) > 0$ and $\alpha (x,x^*) < 0$, it follows from the above inequality

69 $$\begin{aligned} \sum _{i=1}^{p}u^{*}_{i}[f_{i}(x) - \lambda ^{*}_{i}g_{i}(x)] \geqq 0. \end{aligned}$$

Therefore, we conclude that $x^{*}$ is an efficient solution of (*P*).

(b) Using the same idea in (a) and assumption (60), the proof follows.

(c) Let *x* be an arbitrary feasible solution of (*P*). From (60) we observe that

70 $$\begin{aligned}&\frac{1}{\beta (x,x^{*})}\left\langle \sum _{i=1}^{p}u^{*}_{i}\left[ \mathbb {E}\left[ \nabla f_{i}(x^{*})\right] - \lambda ^{*}_{i} \mathbb {E}\left[ \nabla g_{i}(x^{*})\right] \right] + \sum _{m=1}^{\nu _{0}}v^{*}_{m}\mathbb {E}\left[ \nabla G_{j_{m}}(x^{*},t^{m})\right] \right. \nonumber \\&\quad \left. + \sum _{m=\nu _{0} + 1}^{\nu }v^{*}_{m} \mathbb {E}\left[ \nabla H_{k_{m}}(x^{*},s^{m})\right] ,e^{\beta (x,x^{*})\eta (x,x^*)} - \mathbf {1}\right\rangle = 0, \end{aligned}$$

which in view of our $HA(\alpha ,\beta ,\gamma ,\xi ,\eta ,\rho ,h(\cdot ,\cdot ,\omega ),\kappa (\cdot ,\cdot ,\omega ),\psi (\cdot ,\cdot ,\omega ),\varpi (\cdot ,\cdot ,\omega ),\theta )$-random V-asymptotic-pseudoinvexity assumption implies that

71 $$\begin{aligned} \frac{1}{\alpha (x,x^{*})}\gamma (x,x^{*})\left( e^{\alpha (x,x^{*})\sum _{i=1}^{p}\xi _{i}(x,x^{*})[L_{i}(x, u^{*},v^{*},\lambda ^{*},\bar{t},\bar{s}) - L_{i}(x^{*}, u^{*},v^{*},\lambda ^{*},\bar{t},\bar{s})]} - 1\right) \geqq 0. \end{aligned}$$

We need to consider two cases: $\alpha (x,x^{*}) > 0$ and $\alpha (x,x^{*}) < 0$. If we assume that $\alpha (x,x^{*}) > 0$ and recall that $\gamma (x,x^{*}) > 0$, then the above inequality becomes

72 $$\begin{aligned} e^{\alpha (x,x^{*})\sum _{i=1}^{p}\xi _{i}(x,x^{*})[L_{i}(x, u^{*},v^{*},\lambda ^{*},\bar{t},\bar{s}) - L_{i}(x^{*}, u^{*},v^{*},\lambda ^{*},\bar{t},\bar{s})]} \geqq 1, \end{aligned}$$

which implies that

73 $$\begin{aligned} \sum _{i=1}^{p}\xi _{i}(x,x^{*})L_{i}(x, u^{*},v^{*},\lambda ^{*},\bar{t},\bar{s}) \geqq \sum _{i=1}^{p}\xi _{i}(x,x^{*})L_{i}(x^{*}, u^{*},v^{*},\lambda ^{*},\bar{t},\bar{s}). \end{aligned}$$

Because $x^{*} \in \mathbb {F},\; t^{m} \in \hat{T}_{j_{m}}(x^{*}), m \in \underline{\nu _{0}}$, and $\lambda ^{*}_{i} = \varphi _{i}(x^{*}),\; i \in \underline{p}$, the right-hand side of the above inequality is equal to zero, and hence we have $L(x,u^{*},v^{*},\lambda ^{*},\bar{t},\bar{s}) \geqq 0$. Next, as $x \in \mathbb {F},$ and $v^{*}_{m}> 0$, $m \in \underline{\nu _{0}}$, this inequality simplifies to

74 $$\begin{aligned} \sum _{i=1}^{p}u^{*}_{i}\xi _{i}(x,x^{*})[f_{i}(x) - \lambda ^{*}_{i}g_{i}(x)] \geqq 0. \end{aligned}$$

Since $u^{*} > 0$ and $\xi _{i}(x,x^{*}) > 0,\; i \in \underline{p}$, the above inequality implies that

75 $$\begin{aligned} \left( f_{1}(x) - \lambda ^{*}_{1}g_{1}(x), \ldots , f_{p}(x) - \lambda ^{*}_{p}g_{p}(x)\right) \nleqslant (0, \ldots , 0), \end{aligned}$$

which in turn implies that

76 $$\begin{aligned} \left( \frac{f_{1}(x)}{g_{1}(x)},\ldots , \frac{f_{p}(x)}{g_{p}(x)}\right) \nleqslant (\lambda ^{*}_{1},\ldots ,\lambda ^{*}_{p}) = \varphi (x^{*}). \end{aligned}$$

Since $$x \in \mathbb {F}$$ was arbitrary, we conclude from this inequality that $$x^{*}$$ is an efficient solution of (*P*). On the other hand, we arrive at the same conclusion if we assume that $$\alpha (x,x^{*}) < 0.$$$$\square$$

## Concluding remarks

In this paper, we have introduced several notions of random exponential type $HA(\alpha ,\beta ,\gamma ,\xi ,\eta ,\rho , h(\cdot ,\cdot ,\cdot ),\theta )$-V-asymptotic invexities (which generalize the Hanson–Antczak type $(\alpha ,\beta ,\gamma ,\xi ,\eta ,\rho ,h(\cdot ,\cdot ),\theta )$-V-invexity (Zalmai 2013a), while this generalizes most of the existing notions in the literature), and then applied to establish some results to the context of a class of asymptotically sufficient efficiency conditions in semi-infinite multi-objective fractional programming. Furthermore, the obtained results can be applied to generalize the related duality models and theorems in Zalmai (2013b, c), and more. Our results also indicate a wide range of future applications to other problems arising from higher order random invexities and its variants.
